# A Frailty Instrument for primary care: findings from the Survey of Health, Ageing and Retirement in Europe (SHARE)

**DOI:** 10.1186/1471-2318-10-57

**Published:** 2010-08-24

**Authors:** Roman Romero-Ortuno, Cathal D Walsh, Brian A Lawlor, Rose Anne Kenny

**Affiliations:** 1 Department of Medical Gerontology (Trinity College Dublin), Trinity Centre for Health Sciences, St James's Hospital, James's Street, Dublin 8, Ireland; 2 Department of Statistics, Trinity College Dublin, Dublin 2, Ireland; 3 Department of Psychiatry (Trinity College Dublin), Trinity Centre for Health Sciences, St James's Hospital, James's Street, Dublin 8, Ireland; 4 Centre of Excellence for Successful Ageing, St James's Hospital, James's Street, Dublin 8, Ireland; 5 Trinity College Institute of Neuroscience, Trinity College Dublin, Dublin 2, Ireland

## Abstract

**Background:**

A frailty paradigm would be useful in primary care to identify older people at risk, but appropriate metrics at that level are lacking. We created and validated a simple instrument for frailty screening in Europeans aged ≥50. Our study is based on the first wave of the Survey of Health, Ageing and Retirement in Europe (SHARE, http://www.share-project.org), a large population-based survey conducted in 2004-2005 in twelve European countries.

**Methods:**

*Subjects*: SHARE Wave 1 respondents (17,304 females and 13,811 males). *Measures*: five SHARE variables approximating Fried's frailty definition. *Analyses *(for each gender): 1) estimation of a discreet factor (DFactor) model based on the frailty variables using LatentGOLD^®^. A single DFactor with three ordered levels or latent classes (i.e. non-frail, pre-frail and frail) was modelled; 2) the latent classes were characterised against a biopsychosocial range of Wave 1 variables; 3) the prospective mortality risk (unadjusted and age-adjusted) for each frailty class was established on those subjects with known mortality status at Wave 2 (2007-2008) (11,384 females and 9,163 males); 4) two web-based calculators were created for easy retrieval of a subject's frailty class given any five measurements.

**Results:**

*Females*: the DFactor model included 15,578 cases (standard *R*^2 ^= 0.61). All five frailty indicators discriminated well (*p *< 0.001) between the three classes: non-frail (*N *= 10,420; 66.9%), pre-frail (*N *= 4,025; 25.8%), and frail (*N *= 1,133; 7.3%). Relative to the non-frail class, the age-adjusted Odds Ratio (with 95% Confidence Interval) for mortality at Wave 2 was 2.1 (1.4 - 3.0) in the pre-frail and 4.8 (3.1 - 7.4) in the frail. *Males*: 12,783 cases (standard *R*^2 ^= 0.61, all frailty indicators had *p *< 0.001): non-frail (*N *= 10,517; 82.3%), pre-frail (*N *= 1,871; 14.6%), and frail (*N *= 395; 3.1%); age-adjusted OR (95% CI) for mortality: 3.0 (2.3 - 4.0) in the pre-frail, 6.9 (4.7 - 10.2) in the frail.

**Conclusions:**

The *SHARE Frailty Instrument *has sufficient construct and predictive validity, and is readily and freely accessible via web calculators. To our knowledge, SHARE-FI represents the first European research effort towards a common frailty language at the community level.

## Background

Frailty is an emerging geriatric syndrome [1], and its associations include falls, disability, morbidity, mortality and excess healthcare costs from consultations, polypharmacy, hospitalisations and institutionalisations [2-5]. Frailty confers loss of independence, vulnerability and impairs the quality of life and psychological well-being of many older people; it also poses an enormous challenge on families, carers and other structures of social care and social support. The prevalence of frailty in community-dwelling older Europeans (65 years and older) varies between 5.8% and 27.3%; in addition, between 34.6% and 50.9% are classified as 'pre-frail' [6]. In the face of the rapid population ageing occurring in Western societies, frailty is set to reach epidemic proportions over the next few decades.

Frailty is an entity recognised by clinicians, with multiple manifestations and with no single symptom being sufficient or essential in its presentation [7]. In part due to its syndromic nature, and despite considerable research efforts in the field, an operational definition of frailty that meets international consensus is still lacking [8-13]. Defining frailty requires a *complex systems *approach [2] and, in general, it is accepted that a good definition should not only capture the *biological*, but also the *psychosocial *correlates of frailty [14]. In addition, it has been suggested that frailty could have a gendered dimension and manifest differently in males and females [15].

Numerous frailty definitions and assessment tools have been developed in clinical practice and research, and this has been the focus of many reviews and comparative studies [3,16-20]. In particular, Fried *et al*.'s *frailty phenotype *[21,22] has achieved international reputation. The method has been extensively validated in the research literature [23-25]; however, a criticism is that it is not readily applicable in routine primary care practice.

The main advantage of Fried's method is that it requires the measurement of only five variables, namely *weight loss*, *exhaustion*, *grip strength*, *walking speed *and *physical activity *[21]. Whilst this is affordable from a primary care point of view, the problem arises with the construction of the measure. In Fried's definition, frailty is defined in terms of three categories, each of which is defined by the sum of the number of individual criteria present (0: *non-frail*, 1 or 2: *pre-frail*, and 3, 4 or 5: *frail*). The dichotomisation of individual criteria that are measured on a continuous scale (i.e. grip strength, walking speed and physical activity) is done retrospectively according to the lowest twentieth percentile rule, and there are further stratifications. This requires considerable statistical expertise and also a reference sample, both of which are not always available to primary care practitioners.

With the ageing of the population in Western societies and the rising costs of health and social care, many countries are refocusing health policy on health promotion and disability prevention among older people. It has been argued that efforts aimed at identifying at-risk groups of older people in order to provide early intervention and/or multidisciplinary case management should be done *at the level of general practice *via adoption of a clinical paradigm based on the concept of frailty, which fits well with the biopsychosocial model of primary care [26]. However, this ideal has exposed the lack of frailty metrics that are appropriate for primary care. Indeed, family physicians and community practitioners are still in need of easy instruments for frailty [27].

Recently, Santos-Eggimann *et al. *employed an approach to Fried's method in the first wave of the Survey of Health, Ageing and Retirement in Europe (SHARE), in order to establish the prevalence of frailty in middle-aged and older community-dwelling Europeans living in ten countries [6]. Since SHARE did not collect Fried's criteria according to their original definition, Santos-Eggimann *et al. *selected the five SHARE variables that in their view were the closest to Fried's variables. Although their selection was not without significant departures from Fried's theoretical model, their effort represented the first-time attempt to operationalise Fried's frailty phenotype in a very large European population-based sample.

Building upon Santos-Eggimann *et al*.'s work, and using methodology previously described by Bandeen-Roche *et al. *on Fried's original variables [22], the aims of this study were to assess whether those five SHARE variables approaching Fried's frailty phenotype had internal validity *on their own *and could be statistically summarised in a single *factor *with three underlying *latent classes *(i.e. non-frail, pre-frail and frail), with appropriate biopsychosocial correlates and predictive validity.

Rather than replicating Fried's paradigm, our aim was to offer *a valid related alternative *to it in the European context, whist taking advantage of the unparalleled data resource made available by SHARE. The ultimate goal was to provide European community practitioners with a simple and valid instrument that offers a pre-calculated, population-representative and gender-specific frailty class, once the five measurements are entered. The *SHARE Frailty Instrument *(SHARE-FI) is intended to facilitate the rapid assessment of frailty in primary care and enhance the communication between the various agencies managing middle-aged and older people in the community.

## Methods

**Figure 1:**
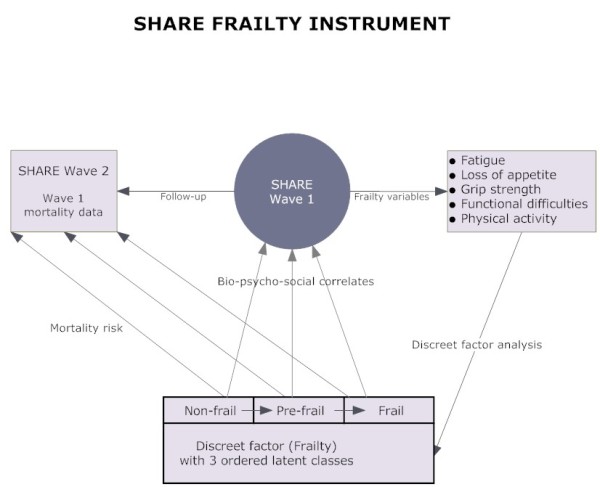
**Development and validation of SHARE-FI (for each gender)**.

An overview of the validation strategy is presented in Figure 1.

### Subjects

17,304 females and 13,811 males included in the first wave of the Survey of Health, Aging and Retirement in Europe (SHARE, release 2.3.0 of November 13th, 2009), corresponding to nationally representative samples of 12 European countries (Austria, Germany, Sweden, Netherlands, Spain, Italy, France, Denmark, Greece, Switzerland, Belgium and Israel).

SHARE aimed at extracting from each country probability samples that would allow inferring from the sample to the finite population of Europeans *aged 50 and over*. The target population of individuals was defined as all individuals born in 1954 or earlier, speaking the official language of the country and not living abroad or in an institution such as a prison (or institution for older people) during the duration of the field work, and their spouses/partners independent of age [28]. For the total pooled sample (*N *= 31,115), country-specific individual response rates ranged between 73.7% and 93.3%, with an average of 85.3% (http://www.share-project.org/t3/share/index.php?id=97, last accessed: August 18^th^, 2010).

Wave 1 data were collected between 2004 and 2006. The mean age (standard deviation) of the females was 63.6 (11.1), and that of males was 64.1 (9.9). Complete data for assessing frailty according to the approach by Santos-Eggimann *et al. *[6] were available for 15,578 females and 12,783 males.

For the prospective validation of SHARE-FI, we used a subset of Wave 1 subjects (11,384 females and 9,163 males) for whom mortality data at Wave 2 (2006 - 2007) were available. Since a considerable proportion (34%) of baseline subjects had missing information on mortality and SHARE could not ascertain the causes of non-response for all respondents [29], we compared (for each gender) subjects with mortality data available *vs*. unavailable, to assess the representativity of the subsamples used for the prospective validation. In addition, we imputed missing mortality data and conducted a *sensitivity analysis *to assess the impact of missing mortality data on the mortality prediction of SHARE-FI. The mean follow up period between Wave 1 and Wave 2 was 2.4 years.

### Frailty definition

All questionnaires, full variable definitions and original variable codes can be found in the SHARE methodology book, which is available online [30].

For the frailty definition, we used the SHARE variables previously selected by Santos-Eggimann *et al. *[6]:

• *Exhaustion *was identified as a positive response to the question: "In the last month, have you had too little energy to do the things you wanted to do?". A positive answer (Yes) was re-coded as 1, and No was re-coded as 0.

• The *weight loss *criterion was fulfilled by reporting a "Diminution in desire for food" in response to the question: "What has your appetite been like?" or, in the case of a non-specific or uncodeable response to this question, by responding "Less" to the question: "So, have you been eating more or less than usual?". The presence of the criterion was coded as 1 and its absence as 0.

• *Weakness *was assessed by handgrip strength (Kg) using a dynamometer. Two consecutive measurements were taken from the left and right hands. The highest of the four was selected. This variable was kept continuous.

• *Slowness *was defined as a positive answer to either of the following two items: "Because of a health problem, do you have difficulty [expected to last more than 3 months] walking 100 metres?" or "... climbing one flight of stairs without resting?". One or two positive answers received the score of 1, and two negative answers received the score of 0.

• The *low activity *criterion was assessed by the question: "How often do you engage in activities that require a low or moderate level of energy such as gardening, cleaning the car, or doing a walk?". This variable was kept ordinal: 1 = "More than once a week"; 2 = "Once a week"; 3 = One to three times a month" and 4 = "Hardly ever or never".

We agree with Santos-Eggimann *et al. *[6] that, among all the items available in SHARE for the total sample, the above choice of variables is the closest possible *vis-à-vis *the original variables in Fried's phenotype [21]. However, we acknowledge two significant departures from Fried's theoretical framework, namely as regards 'weight loss' (replaced by appetite) and 'slowness' (measured by questions on functional limitation). Therefore, the aim was not to validate SHARE-FI *vis-à-vis *Fried's phenotype, but to establish the validity of SHARE-FI *on its own*, whilst acknowledging its sources.

### Measures for cross-sectional correlations

#### Sociodemographic domain

• *Age *(years). It was obtained by subtracting the year of birth from 2004.

• *Years of education*.

#### Physical domain

• *Self-rated health*: excellent (1), very good (2), good (3), fair (4), or poor (5).

• *Number of chronic diseases*: calculated as the sum of affirmative self-reports to the following conditions (if diagnosed by a doctor): 1. Heart attack or myocardial infarction or coronary thrombosis or any other heart problem including congestive heart failure; 2. High blood pressure or hypertension; 3. High blood cholesterol; 4. Stroke or cerebral vascular disease; 5. Diabetes or high blood sugar; 6. Chronic lung disease such as chronic bronchitis or emphysema; 7. Asthma; 8. Arthritis, including osteoarthritis, or rheumatism; 9. Osteoporosis; 10. Cancer or malignant tumour, including leukaemia or lymphoma, but excluding minor skin cancers; 11. Stomach or duodenal ulcer, peptic ulcer; 12. Parkinson disease; 13. Cataracts; 14. Hip fracture or femoral fracture; 15. Other fractures; 16. Alzheimer's disease, dementia or senility; 17. Benign tumour and 18. Other conditions.

• *Number of medical symptoms *present for at least the past six months.

• *Number of contacts with a medical doctor *in the past 12 months.

• *Admitted to hospital *in the past 12 months (yes or no).

#### Functional domain

• *Number of limitations with activities of daily living*.

• *Number of limitations with instrumental activities of daily living*.

• *Received home care for personal or nursing care *in the last 12 months: yes or no.

• *Received home care for domestic tasks *in the last 12 months: yes or no.

#### Psychological and cognitive domains

• *EURO-D *depression scale [31].

• *Verbal fluency test *score: maximum number of different animals that the respondent is able to name in 60 seconds.

• *Delayed word recall*: maximum number of words (out of a list of 10) that the respondent is able to recall after an initial recall followed by the verbal fluency and numeracy tests.

### Mortality measures (for prospective validation)

The release 2.3.0 of Wave 1 contains a variable that informs whether Wave 1 respondents were still alive in Wave 2, deceased between Wave 1 and Wave 2, or with mortality information unavailable. Information was available for 11,384 females and 9,163 males (66% of the baseline sample).

### Statistical analyses

#### Univariate descriptives

Individual variable descriptives were given as mean and standard deviation (SD) or proportion (percentage) as appropriate.

#### Level of significance

It was set at 0.01 throughout.

#### Estimation of a discreet factor (DFactor) model

For each gender, a DFactor model was estimated based on the five SHARE frailty variables using the LatentGOLD^® ^package (version 4.5.0) http://www.statisticalinnovations.com. A single DFactor with three ordered levels or latent classes (non-frail, pre-frail and frail) was requested. To assess the extent to which the frailty variables discriminated between the classes independently of age, each DFactor model was repeated with age as covariate.

In the context of this study, DFactor analysis is preferable to traditional Factor Analysis (FA) because in DFactor analysis the observed variables may be of mixed scale types including nominal (i.e. *exhaustion*, *weight loss *and *slowness*), ordinal (i.e. *low activity*) and continuous (i.e. *weakness*). The latent variables are not continuous but discrete, in our case containing 3 ordered categories (levels). Importantly, in DFactor analysis the model is not linear.

For each gender, the non age-adjusted DFactor model classification was saved in SPSS 16.0 format and merged with SHARE Wave 1 data to allow for correlations between the classes and various biopsychosocial variables, as well as with the mortality at Wave 2 variable.

#### Correlations of the latent classes against SHARE Wave 1 variables

The two-tailed Spearman's rank correlation (*rho*) coefficient was used to correlate the (ordinal) latent class membership variable against continuous and ordinal variables; the SPSS partial correlations procedure was used to control for the effects of age in these correlations. The Chi-squared for trend was used to test the association between the latent class membership variable and dichotomous variables; to control for the effects of age, ordinal regression (with latent class as dependent variable) was used.

#### Prospective validation of the DFactor

Binary logistic regression was conducted to assess whether the DFactor classes at Wave 1 significantly predicted whether or not a subject was dead by Wave 2. In the model, the latent class membership variable was entered as a categorical predictor, using the non-frail class as reference category, and simple contrasts were requested. The dependent variable (i.e. dead at Wave 2, coded 0 = no and 1 = yes) included non-missing data only. The odds ratio (OR) for mortality was indicated by the Exp(B) statistic in the binary logistic regression model. Ninety-five percent confidence intervals for ORs were requested. Age-adjusted models were also computed.

#### Sensitivity analysis for the prospective validation

For each gender, comparisons between mortality-available and mortality-unavailable groups were conducted with the Chi-squared test (for dichotomous variables) or the Mann-Whitney U test (for continuous or ordinal variables).

For the sensitivity analysis, the imputation of missing mortality data was conducted, for each gender and frailty class, as follows: we counted the number of cases who were not lost to follow up (B), and then we established the number cases who died (A). The unadjusted mortality rate (A/B%) was defined as the expected mortality rate for the missing cases and was randomly applied to them. The rest of the missing cases were coded as alive.

#### Construction of the SHARE-FI calculators

For each gender, this involved the following steps: 1) use of the individual variable loadings on the DFactor to derivate a linear formula for the calculation of the predicted DFactor score; 2) use of SPSS curve estimation procedures to understand the non-linear relationship between predicted and empirical DFactor scores; 3) identification of the two latent class cut-off scores on the non-linear fitted curve, and 4) incorporation of the predicted DFactor score formula and class cut-off values into a web-based interface that, following entry of the five frailty measurements, automatically calculates the score and adjudicates its class.

The formula for the predicted DFactor score is obtained by considering the coefficients of the individual variables on the DFactor (as shown in LatentGOLD^® ^output). In order to construct the raw score, the tool uses a linear combination of the standardised variables that have been entered in the calculator. The raw frailty score for a new individual, *i*, can be expressed as follows:

$$\text{DFactor score(i)}={\text{z}}_{\text{fat}}{\text{w}}_{\text{fat}}\text{(i)}+{\text{z}}_{\text{loss}}{\text{w}}_{\text{loss}}\text{(i)}+{\text{z}}_{\text{grip}}{\text{w}}_{\text{grip}}\text{(i)}+{\text{z}}_{\text{fdiff}}{\text{w}}_{\text{fdiff}}\text{(i)}+{\text{z}}_{\text{act}}{\text{w}}_{\text{act}}\text{(i),}$$

where FAT is fatigue, LOSS is loss of appetite, GRIP is grip strength, FDIFF is functional difficulties and ACT is physical activity, as defined in the above frailty definition. The value z_fat_(i) represents the standardised score on the fatigue measure for the individual *i*, and similarly for the other standardised scores. The w_fat _is the weighting associated with that measure on the DFactor and is obtained from the LatentGOLD^® ^output.

Once the predicted DFactor score formula and class cut-off values were obtained, an interface was designed using Microsoft Office Excel 2007. The spreadsheet was then converted to a webpage using the commercially available SpreadsheetConverter to HTML/JavaScript^® ^version 4.5.2 (Framtidsforum I & M AB, Uppsala Sweden, http://www.spreadsheetconverter.com).

## Results

Table 1 summarises the SHARE-FI validation results.

**Table 1:** Frailty latent classes by gender: cross-sectional correlates and mortality prediction.

	Females (*N *= 15,578)					Males (*N *= 12,783)				
	**Non-frail** **(*N *= 10,420)**	**Pre-frail** **(*N *= 4,025)**	**Frail** **(*N *= 1,133)**	***P *value**	**Age-adjusted *P *value**	**Non-frail** **(*N *= 10,517)**	**Pre-frail** **(*N *= 1,871)**	**Frail** **(*N *= 395)**	***P *value**	**Age-adjusted *P *value**

**Frailty phenotype: DFactor model**										

Exhaustion: yes (cp)	0.21	0.51	0.81	**<0.001**^Ω^	**<0.001**^Ω^	0.17	0.47	0.80	**<0.001**^Ω^	**<0.001**^Ω^

Weight loss: yes (cp)	0.03	0.12	0.39	**<0.001**^Ω^	**<0.001**^Ω^	0.03	0.12	0.42	**<0.001**^Ω^	**<0.001**^Ω^

Handgrip strength: mean (Kg)	29.4	23.7	17.9	**<0.001**^Ω^	**<0.001**^Ω^	45.8	36.2	26.5	**<0.001**^Ω^	**<0.001**^Ω^

Slowness: yes (cp)	0.02	0.22	0.84	**<0.001**^Ω^	**<0.001**^Ω^	0.01	0.29	0.92	**<0.001**^Ω^	**<0.001**^Ω^

Low activity: hardly ever, or never (cp)	0.03	0.17	0.54	**<0.001**^Ω^	**<0.001**^Ω^	0.03	0.21	0.62	**<0.001**^Ω^	**<0.001**^Ω^

**Sociodemographic**										

Age: mean (SD)	60.5 (9.4)	66.7 (10.9)	73.3 (11.0)	**<0.001**^#^	-	62.6 (9.0)	69.1 (10.4)	74.0 (10.2)	**<0.001**^#^	-

Years of education: mean (SD)	10.3 (4.2)	8.2 (4.4)	6.5 (4.4)	**<0.001**^#^	**<0.001**^§^	10.9 (4.3)	8.6 (4.7)	7.8 (5.0)	**<0.001**^#^	**<0.001**^§^

**Physical**										

Self-rated health (best:1; worst:5): mean (SD)	2.7 (1.0)	3.5 (0.9)	4.2 (0.8)	**<0.001**^#^	**<0.001**^§^	2.7 (1.0)	3.7 (1.0)	4.4 (0.7)	**<0.001**^#^	**<0.001**^§^

No. chronic diseases: mean (SD)	1.2 (1.2)	2.1 (1.5)	3.1 (1.8)	**<0.001**^#^	**<0.001**^§^	1.2 (1.2)	2.1 (1.5)	2.9 (1.7)	**<0.001**^#^	**<0.001**^§^

No. symptoms: mean (SD)	1.2 (1.3)	2.3 (1.8)	3.9 (2.3)	**<0.001**^#^	**<0.001**^§^	0.9 (1.1)	2.0 (1.7)	3.4 (2.1)	**<0.001**^#^	**<0.001**^§^

No. contacts with doctor in last year: mean (SD)	5.0 (7.1)	9.4 (11.4)	15.5 (17.0)	**<0.001**^#^	**<0.001**^§^	4.9 (7.6)	10.1 (12.4)	17.5 (20.2)	**<0.001**^#^	**<0.001**^§^

Admitted to hospital in last year (%)	7.9	16.5	30.0	**<0.001**^χ^	**<0.001**^Δ^	10.4	23.5	36.7	**<0.001**^χ^	**<0.001**^Δ^

**Functional**										

No. limitations with ADLs: mean (SD)	0.0 (0.2)	0.2 (0.7)	1.1 (1.5)	**<0.001**^#^	**<0.001**^§^	0.0 (0.3)	0.3 (0.9)	1.3 (1.7)	**<0.001**^#^	**<0.001**^§^

No. limitations with IADLs: mean (SD)	0.1 (0.3)	0.5 (1.0)	1.8 (1.8)	**<0.001**^#^	**<0.001**^§^	0.1 (0.3)	0.5 (1.1)	1.9 (2.0)	**<0.001**^#^	**<0.001**^§^

Received home care: personal/nursing (%)	1.9	5.9	18.6	**<0.001**^χ^	**<0.001**^Δ^	1.6	6.8	17.2	**<0.001**^χ^	**<0.001**^Δ^

Received home care: domestic tasks (%)	1.9	10.3	28.7	**<0.001**^χ^	**<0.001**^Δ^	1.0	6.9	19.2	**<0.001**^χ^	**<0.001**^Δ^

**Psychological and cognitive**										

EURO-D score (min: 0; max: 12) (SD)	1.9 (1.8)	3.7 (2.4)	5.5 (2.6)	**<0.001**^#^	**<0.001**^§^	1.4 (1.6)	3.1 (2.3)	5.2 (2.7)	**<0.001**^#^	**<0.001**^§^

Verbal fluency test score: mean (SD)	20.2 (7.1)	16.5 (6.7)	13.1 (6.0)	**<0.001**^#^	**<0.001**^§^	20.1 (7.1)	15.9 (6.4)	13.2 (5.7)	**<0.001**^#^	**<0.001**^§^

Delayed word recall score: mean (SD)	3.9 (2.0)	3.0 (2.0)	1.9 (1.8)	**<0.001**^#^	**<0.001**^§^	3.4 (1.9)	2.5 (1.8)	1.7 (1.6)	**<0.001**^#^	**<0.001**^§^

**Mortality at Wave 2**										

% dead	0.7	2.6	9.2	**<0.001**^χ^	**<0.001**^Δ^	2.0	8.8	22.6	**<0.001**^χ^	**<0.001**^Δ^

OR for mortality (95% CI)	1.0	3.7 (2.5-5.3)	14.2 (9.7-20.8)	-	-	1.0	4.8 (3.7-6.2)	14.6 (10.3-20.7)	-	-

Age-adjusted OR for mortality (95% CI)	1.0	2.1 (1.4- 3.0)	4.8 (3.1- 7.4)	-	-	1.0	3.0 (2.3-4.0)	6.9 (4.7-10.2)	-	-

cp: conditional probability (from the DFactor model profile output); SD: standard deviation; ADLs: (basic) activities of daily living; IADLs: instrumental activities of daily living; OR: odds ratio; CI: confidence interval. Significant *P *values (<0.01) are indicated in bold.

^Ω ^Wald statistic; ^# ^two-tailed Spearman's *rho *test; ^§ ^Partial correlation; ^χ ^Chi-squared for trend; ^Δ ^ordinal regression.

### Estimation of the DFactor models

#### DFactor for females

The DFactor model included 15,578 cases. Fifteen parameters were estimated, standard *R*^2 ^= 0.61, entropy *R*^2 ^= 0.43. All five frailty indicators discriminated well (*p *< 0.001) between the three classes: non-frail (*N *= 10,420; 66.9%), pre-frail (*N *= 4,025; 25.8%), and frail (*N *= 1,133; 7.3%). The DFactor loadings (with *R*^2 ^representing the communality of the indicator) were:

• *Fatigue *= 0.4088 (*R*^2 ^= 0.1671)

• *Loss of appetite *= 0.3325 (*R*^2 ^= 0.1246)

• *Grip strength *= -0.4910 (*R*^2 ^= 0.2411)

• *Functional difficulties *= 0.6012 (*R*^2 ^= 0.4109)

• *Physical activity *= 0.4818 (*R*^2 ^= 0.2445)

The conditional probabilities (given class membership) for the non-continuous indicators and the mean grip strength for each latent class are shown in Table 1. Entering age as a covariate in the DFactor model did not significantly change the results (standard *R*^2 ^= 0.67, entropy *R*^2 ^= 0.52, all indicators with *p *< 0.001). The age-adjusted *R*^2 ^communalities were: *Fatigue *= 0.1057, *Loss of appetite *= 0.0818, *Grip strength *= 0.4159, *Functional difficulties *= 0.3444 and *Physical activity *= 0.1731.

#### DFactor for males

The DFactor model included 12,783 cases. Fifteen parameters were estimated, standard *R*^2 ^= 0.61, entropy *R*^2 ^= 0.47. All five frailty indicators discriminated well (*p *< 0.001) between the three classes: non-frail (*N *= 10,517; 82.3%), pre-frail (*N *= 1,871; 14.6%), and frail (*N *= 395; 3.1%). The DFactor loadings were:

• *Fatigue *= 0.3762 (*R*^2 ^= 0.1415)

• *Loss of appetite *= 0.3130 (*R*^2 ^= 0.1133)

• *Grip strength *= -0.4653 (*R*^2 ^= 0.2165)

• *Functional difficulties *= 0.6146 (*R*^2 ^= 0.4075)

• *Physical activity *= 0.4680 (*R*^2 ^= 0.2244)

The conditional probabilities (given class membership) for the non-continuous indicators and the mean grip strength for each latent class are shown in Table 1. Entering age as a covariate in the DFactor model did not significantly change the results (standard *R*^2 ^= 0.70, entropy *R*^2 ^= 0.57, all indicators with *p *< 0.001). The age-adjusted *R*^2 ^communalities were: *Fatigue *= 0.0627, *Loss of appetite *= 0.0501, *Grip strength *= 0.5342, *Functional difficulties *= 0.2206 and *Physical activity *= 0.0874.

### Bivariate correlations

As Table 1 shows, all the correlations (unadjusted and age-adjusted) were statistically significant (*p *< 0.001), and of moderate effect size [32]. For example, in females, the unadjusted/age-adjusted correlation coefficients were: 0.46/0.41 for self-rated health, 0.38/0.32 for chronic diseases, 0.40/0.40 for symptoms, 0.32/0.28 for visits to the doctor, 0.35/0.34 for ADL limitations, 0.42/0.41 for IADL limitations, and 0.45/0.48 for EURO-D.

In males, the unadjusted/age-adjusted correlation coefficients were: 0.40/0.37 for self-rated health, 0.28/0.24 for chronic diseases, 0.32/0.36 for symptoms, 0.28/0.26 for visits to the doctor, 0.31/0.36 for ADL limitations, 0.39/0.41 for IADL limitations, and 0.36/0.44 for EURO-D.

### Predictive validity of SHARE-FI

Table 1 shows, for females and males, the unadjusted and age-adjusted odds ratios (ORs) for mortality at Wave 2 (with 95% confidence intervals, CIs) given the frailty class in Wave 1.

In females, relative to the non-frail class, the unadjusted OR was 3.7 (2.5-5.3) in the pre-frail and 14.2 (9.7-20.8) in the frail. The age-adjusted OR was 2.1 (1.4 - 3.0) in the pre-frail and 4.8 (3.1 - 7.4) in the frail.

In males, relative to the non-frail class, the unadjusted OR was 4.8 (3.7 - 6.2) in the pre-frail and 14.6 (10.3 - 20.7) in the frail. The age-adjusted OR was 3.0 (2.3 - 4.0) in the pre-frail and 6.9 (4.7 - 10.2) in the frail.

Tables 2 and 3 show the comparisons between mortality-available and mortality-unavailable subgroups. *In females*, the subgroup with missing mortality data (*N *= 5,920) had significantly higher levels of frailty in all but one (i.e. exhaustion) frailty parameters; they were more likely to be pre-frail and frail and less likely to be non-frail; they had worse self-rated health, higher number of contacts with the doctor in the past year, more limitations with IADLs and a worse cognitive profile (Table 2).

**Table 2:** Comparison of baseline characteristics between Wave 1 subjects with and without mortality data available (females).

	Mortality data available (*N *= 11,384)	Mortality data not available (*N *= 5,920)	Significance of the difference (*P*)
**Frailty variables**			

Exhaustion: yes (%)	36.1	37.0	0.244^χ^

Weight loss: yes (%)	9.1	11.5	**< 0.001**^χ^

Handgrip strength, Kg: mean (SD)	26.9 (7.4)	26.1 (7.8)	**< 0.001**^μ^

Slowness: yes (%)	16.7	19.3	**< 0.001**^χ^

Low activity: hardly ever, or never (%)	12.3	18.1	**< 0.001**^χ^

**Frailty classes**			

Non-frail (%)	68.5	63.6	**< 0.001**^χ^

Pre-frail (%)	25.0	27.6	**0.001**^χ^

Frail (%)	6.5	8.9	**< 0.001**^χ^

**Sociodemographic**			

Age: mean (SD)	63.6 (10.9)	63.8 (11.4)	0.627^μ^

Years of education: mean (SD)	9.4 (4.5)	9.3 (4.4)	0.499^μ^

**Physical**			

Self-rated health (best:1; worst:5): mean (SD)	3.0 (1.1)	3.1 (1.1)	**< 0.001**^μ^

No. chronic diseases: mean (SD)	1.6 (1.5)	1.6 (1.5)	0.057^μ^

No. symptoms: mean (SD)	1.7 (1.7)	1.7 (1.8)	0.057^μ^

No. contacts with doctor in last year: mean (SD)	6.8 (9.7)	8.3 (11.9)	**< 0.001**^μ^

Admitted to hospital in last year (%)	12.4	12.9	0.287^χ^

**Functional**			

No. limitations with ADLs: mean (SD)	0.22 (0.80)	0.27 (0.93)	0.054^μ^

No. limitations with IADLs: mean (SD)	0.39 (1.08)	0.50 (1.22)	**< 0.001**^μ^

Received home care: personal/nursing (%)	5.1	4.4	0.046^χ^

Received home care: domestic tasks (%)	6.5	7.5	0.023^χ^

**Psychological and cognitive**			

EURO-D score (min: 0; max: 12) (SD)	2.7 (2.3)	2.8 (2.5)	0.057^μ^

Verbal fluency test score: mean (SD)	18.8 (7.3)	17.9 (7.6)	**< 0.001**^μ^

Delayed word recall score: mean (SD)	3.6 (2.1)	3.2 (2.0)	**< 0.001**^μ^

^χ ^Chi-squared test; ^μ ^Mann-Whitney *U *test. Significant *P *values (< 0.01) are indicated in bold.

**Table 3:** Comparison of baseline characteristics between Wave 1 subjects with and without mortality data available (males).

	Mortality data available (*N *= 9,163)	Mortality data not available (*N *= 4,648)	Significance of the difference (*P*)
**Frailty variables**			

Exhaustion: yes (%)	25.3	27.4	**0.007**^χ^

Weight loss: yes (%)	5.9	7.8	**< 0.001**^χ^

Handgrip strength, Kg: mean (SD)	43.6 (10.5)	42.4 (11.5)	**< 0.001**^μ^

Slowness: yes (%)	10.9	13.6	**< 0.001**^χ^

Low activity: hardly ever, or never (%)	8.5	14.6	**< 0.001**^χ^

**Frailty classes**			

Non-frail (%)	83.9	79.0	**< 0.001**^χ^

Pre-frail (%)	13.5	17.0	**< 0.001**^χ^

Frail (%)	2.7	4.0	**< 0.001**^χ^

**Sociodemographic**			

Age: mean (SD)	64.2 (9.7)	64.0 (10.1)	0.071^μ^

Years of education: mean (SD)	10.4 (4.5)	10.5 (4.5)	0.018^μ^

**Physical**			

Self-rated health (best:1; worst:5): mean (SD)	2.9 (1.1)	3.0 (1.2)	**< 0.001**^μ^

No. chronic diseases: mean (SD)	1.4 (1.3)	1.5 (1.4)	0.046^μ^

No. symptoms: mean (SD)	1.2 (1.4)	1.3 (1.5)	0.080^μ^

No. contacts with doctor in last year: mean (SD)	5.7 (9.0)	7.4 (11.9)	**< 0.001**^μ^

Admitted to hospital in last year (%)	13.1	14.9	**0.003**^χ^

**Functional**			

No. limitations with ADLs: mean (SD)	0.16 (0.66)	0.22 (0.90)	**0.004**^μ^

No. limitations with IADLs: mean (SD)	0.23 (0.89)	0.34 (1.07)	**< 0.001**^μ^

Received home care: personal/nursing (%)	3.3	3.4	0.974^χ^

Received home care: domestic tasks (%)	2.7	3.4	0.058^χ^

**Psychological and cognitive**			

EURO-D score (min: 0; max: 12) (SD)	1.8 (1.9)	2.0 (2.2)	**< 0.001**^μ^

Verbal fluency test score: mean (SD)	19.3 (7.3)	18.6 (7.5)	**< 0.001**^μ^

Delayed word recall score: mean (SD)	3.2 (1.9)	3.0 (1.9)	**< 0.001**^μ^

^χ ^Chi-squared test; ^μ ^Mann-Whitney *U *test. Significant *P *values (< 0.01) are indicated in bold.

*In males*, the subgroup with missing mortality data (*N *= 4,648) had significantly higher levels of frailty in all frailty parameters; they were more likely to be pre-frail and frail and less likely to be non-frail; they had worse self-rated health, higher number of contacts with the doctor in the past year, more hospital admissions in the last year, more limitations with ADLs and IADLs, and a worse psychological and cognitive profile (Table 3).

#### Sensitivity analysis

Table 4 shows the cross-tabulation for the imputation of missing mortality status, by frailty class and gender. Based on this, for females and males, we recalculated the unadjusted and age-adjusted odds ratios (ORs) for mortality at Wave 2 (with 95% confidence intervals, CIs) given the frailty class in Wave 1:

**Table 4:** Cross-tabulation for imputation of missing mortality status, by frailty class and gender.

	Females						Males					
**Frailty class**	***N *(class)**	***N*** **(mortality available)**	***N*** **(died)**	**Unadj. mortality rate (%)**	***N *(mortality missing)**	***N *missing expected as dead**	***N *(class)**	***N*** **(mortality available)**	***N*** **(died)**	**Unadj. mortality rate (%)**	***N *(mortality missing)**	***N *missing expected as dead**

Non-frail	10,420	7,179	51	0.7	3,241	23	10,517	7,231	142	2.0	3,286	66

Pre-frail	4,025	2,621	67	2.6	1,404	37	1,871	1,162	102	8.8	709	62

Frail	1,133	682	63	9.2	451	41	395	230	52	22.6	165	37

*In females*, relative to the non-frail class, the unadjusted OR was 3.7 (2.7 - 5.0) in the pre-frail and 14.1 (10.4 - 19.2) in the frail. The age-adjusted OR was 2.5 (1.9 - 3.5) in the pre-frail and 6.9 (4.9 - 9.7) in the frail.

*In males*, relative to the non-frail class, the unadjusted OR was 4.8 (3.9 - 5.9) in the pre-frail and 14.4 (11.0 - 18.9) in the frail. The age-adjusted OR was 3.8 (3.1 - 4.8) in the pre-frail and 10.0 (7.4 - 13.4) in the frail.

### Frailty calculators

Using the procedure described above, two SHARE-FI calculators (one for females and one for males) were produced and converted to HTML format. The calculators are attached to this manuscript as Additional file 1 (females) and Additional file 2 (males).

The final formula for the predicted DFactor score (DFS) in females was:

$$\begin{array}{l} DFS\left(females\right)=\left(2.077707*Fatigue-0.757295\right)*0.4088+(3.341539*Loss\;of\\ appetite-0.332289)*0.3325+\left(0.132827*Grip\;strength-3.534515\right)*-0.4910+\\ \left(2.627085*Functional\;difficulties-0.461808\right)*0.6012+(0.918866*Physical\\ activity-1.523633)*0.4818 \end{array}$$

The predicted DFS formula for males was:

$$\begin{array}{l} DFS\left(males\right)=\left(2.280336*Fatigue-0.592393\right)*0.3762+(4.058274*Loss\;of\\ appetite-0.263501)*0.3130+\left(0.092326*Grip\;strength-3.986646\right)*\;0.4653+\\ \left(3.098226*Functional\;difficulties-0.365971\right)*0.6146+(1.005942*Physical\\ activity-1.571803)*0.4680 \end{array}$$

**Figure 2:**
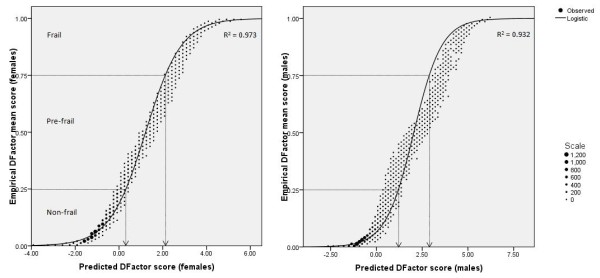
**Selection of predicted DFactor cut-off scores for automatic adjudication of frailty class in the SHARE-FI calculators**.

Figure 2 shows the non-linear relationship between the empirical DFS (as per LatentGOLD^® ^output) and the predicted DFS, for each gender. Logistic curves had the best fit (*R*^2 ^= 0.973 in females, *R*^2 ^= 0.932 in males). Examination of the empirical DFactor mean score against the latent class membership variable revealed that the empirical cut-offs for the frailty classes were 0.25 and 0.75. Extrapolation of these cut-offs to the predicted DFS provided the following cut-offs for the SHARE-FI calculators:

#### Females

• If predicted DFS < 0.3151361243, NON-FRAIL

• If predicted DFS < 2.1301121973, PRE-FRAIL

• If predicted DFS < 6, FRAIL

#### Males

• If predicted DFS < 1.211878526, NON-FRAIL

• If predicted DFS < 3.0052612772, PRE-FRAIL

• If predicted DFS < 7, FRAIL

## Discussion

The aim of this study was to provide European community practitioners with a valid and simple tool to measure the level of frailty in individuals aged ≥50 years given five quick measurements, leading to the obtention of a frailty class relative to a large population-based sample (Wave 1 of SHARE).

In this study, a gendered approach to frailty was adopted and psychosocial correlates of frailty were explored. Two frailty calculators, one for males and one for females, were created and the correlations of the frailty classes against a range of SHARE variables were presented to demonstrate the concurrent criterion validity of SHARE-FI. We also explored the prospective validity of SHARE-FI as a predictor of mortality over a mean follow up of 2.4 years.

The frailty definition used in this study is related (but not equivalent) to the one by Fried *et al. *[21], and was first used by Santos-Eggimann *et al. *[6]. Our aim was not to compare the *construct validity *of this definition *vis-à-vis *Fried's construct, but to offer *a valid related alternative *to it in the European context, whist taking advantage of the unparalleled data resource made available by SHARE.

Both in females and males, SHARE-FI was proven to conform to a discreet, 3-level ordinal latent construct with decreasing population prevalence. The overall prevalence of pre-frailty was 25.8% in females and 14.6% in males; and the prevalence of frailty was 7.3% in females and 3.1% in males. This is highly consistent with the original Cardiovascular Health Study by Fried *et al. *[21], which showed a prevalence of frailty in the original cohort (1989-1990) of 7.3% in females and 4.9% in males.

In addition to its ability to predict mortality, SHARE-FI had *concurrent criterion validity *against a range of measures, as shown by the medium-sized correlations with indices of both subjective (self-rated) and objective (e.g. number of chronic diseases) health, and also with health care utilisation (e.g. number of contacts with a doctor). This would make SHARE-FI not only appealing to family physicians/general practitioners, but also to public health practitioners and health policy makers.

To our knowledge, SHARE-FI represents the first European research effort to facilitate a common language for frailty in the community and the adoption of the clinical frailty paradigm in primary care, both of which were overdue [26,27]. As it currently stands, the main potential use of SHARE-FI is the screening and monitoring of frailty in the community or primary care setting in order to help decide who would benefit from secondary care referrals and/or early multidisciplinary case management. To that effect, the advantages of SHARE-FI are that it can be easily administered in the community by non-physicians (e.g. nurse, health visitor or other allied health professionals), and it is a brief instrument. In view of the prospective mortality results, pre-frail, as well as frail, subjects should be considered, regardless of age, for further assessment.

A limitation of our prospective validation was the significant proportion of missing mortality data in the baseline SHARE Wave 1 sample. The results of the comparison between mortality-available and mortality-unavailable subgroups (Tables 2 and 3) suggest that there was a higher burden of frailty at baseline in those for whom mortality information could not be obtained at follow-up. However, our sensitivity analysis suggested that this could have resulted in *underestimation *of the age-adjusted mortality odds ratios. To assess the extent of this underestimation, further prospective validation of SHARE-FI in other samples is desirable.

Given that SHARE represents the non-institutionalised population aged 50 and older, we recommend using SHARE-FI from the age of 50 onwards. Although the seminal study by Fried *et al. *only included participants aged 65 and older [21], international consensus does not exist on a fixed age cut-off for the definition of frailty, perhaps in recognition that frailty is more about the *biological *age than the *chronological *age of individuals [33]. In any case, all our correlations were significant independently of age (Table 1).

Given its population reference, SHARE-FI would not be appropriate for the screening of frailty in non-community settings (e.g. hospitals or geriatric residential settings), as not only SHARE did not target this population, but the average level of frailty in those settings could make some of the items inapplicable and/or inappropriate. Even in the community, another potential limitation of SHARE-FI is its considerable reliance on self-report, which forms the basis for four out of five items (i.e. all except handgrip strength). In dementia cases, proxy report could be of help, but the answers to the fatigue and appetite items may still be unreliable. In this light, it is not surprising that the latter two items had the lowest communalities in the DFactor models. Cultural influences in the interpretation of these subjective items may also be responsible for the variable prevalences of frailty between European countries previously reported by Santos-Eggimann *et al. *[6]. The methodology described in this paper may be applied to the construction of *country-specific *SHARE-FI calculators, although this approach has the risk of underpower.

## Conclusions

We created and validated the *SHARE Frailty Instrument*, a simple frailty screening instrument for primary care based on the first Wave of the Survey of Health, Ageing and Retirement in Europe. SHARE-FI has sufficient construct and predictive validity, and is readily and freely accessible via web calculators (see Additional file 1 and Additional file 2). To our knowledge, SHARE-FI represents the first European research effort towards a common frailty language at the community level. The Survey for Health, Ageing and Retirement in Europe, in its completed and future waves, is providing, and will continue to provide, excellent opportunities for the scientific study of frailty in Europe.

## Competing interests

The authors declare that they have no competing interests.

## Authors' contributions

RRO conceived the study, performed the statistical analyses, drafted the manuscript and designed the FI calculators. CDW revised the statistical analyses and helped to draft the manuscript. BAL participated in the design of the study and helped to draft the manuscript. RAK participated in the design of the study and helped to draft the manuscript. All authors read and approved the final manuscript.

## Pre-publication history

The pre-publication history for this paper can be accessed here:

http://www.biomedcentral.com/1471-2318/10/57/prepub

## Supplementary Material

Additional file 1 **SHARE Frailty Instrument calculator (females)**. SHARE-FI calculator - females.zip. Click here for file

Additional file 2 **Frailty Instrument calculator (males)**. SHARE-FI calculator - males.zip. Click here for file
