# Supplementary material for: A Frailty Instrument for primary care: findings from the Survey of Health, Ageing and Retirement in Europe (SHARE)
Source: BMC Geriatr. 2010 Aug 24;10:57. doi: 10.1186/1471-2318-10-57 (PMC2939541; doi:10.1186/1471-2318-10-57)
Supplement: Additional file 1 — SHARE Frailty Instrument calculator (females). SHARE-FI calculator - females.zip. [file 1471-2318-10-57-S1.ZIP › SHARE-FI calculator - females/SHARE-FI calculator - females.htm]

SHARE-FI calculator - females.xlsx

| SHARE-FI CALCULATOR: FEMALES |  |  |  |  |
|  |  |  | | |
|  |  |  | | |
| EXHAUSTION |  |  | | |
| In the last month, have you had too little energy to do the things you wanted to do? |  |  | | |
|  | Yes No |  | | |
|  |  |  | | |
|  |  |  | | |
| LOSS OF APPETITE |  |  | | |
| What has your appetite been like? |  |  | | |
|  | Diminution in desire for food and/or eating less than usual No change in desire for food and/or eating the same as usual Increase in desire for food and/or eating more than usual |  | | |
|  |  |  | | |
|  |  |  | | |
| WEAKNESS |  |  | | |
| Maximum grip strength in Kilograms: |  |  | | |
|  |  |  | | |
| Right hand: |  |  | | |
| Attempt 1: |  |  | | |
| Attempt 2: |  |  | | |
| Left hand: |  |  | | |
| Attempt 1: |  |  | | |
| Attempt 2: |  |  | | |
|  |  |  | | |
|  |  |  | | |
| WALKING DIFFICULTIES |  |  | | |
| Because of a health or physical problem, do you have any difficulty doing any of the following everyday activities? | |  | | |
| (Exclude any difficulties that you expect to last less than three months) |  |  | | |
|  |  |  | | |
|  |  |  | | |
| Walking 100 metres: | Yes No |  | | |
|  |  |  | | |
| Climbing one flight of stairs without resting: | Yes No |  | | |
|  |  |  | | |
|  |  |  | | |
|  |  |  | | |
| LOW PHYSICAL ACTIVITY |  |  | | |
| How often do you engage in activities that require a low or moderate level of energy such as gardening, cleaning the car, or doing a walk? | |  | | |
|  |  |  | | |
|  | Hardly ever, or never One to three times a month Once a week More than once a week |  | | |
|  |  |  | | |
|  |  |  | | |
|  |  |  | | |
| FRAILTY SCORE: |  |  | | |
|  |  |  | | |
|  |  |  | | |
| FRAILTY CATEGORY: |  |  | | |


The browser does not support JavaScript. The calculations created using SpreadsheetConverter will not work. Please access the web page using another browser.
