# Supplementary figures and images for: A Frailty Instrument for primary care: findings from the Survey of Health, Ageing and Retirement in Europe (SHARE)
Source: BMC Geriatr. 2010 Aug 24;10:57. doi: 10.1186/1471-2318-10-57 (PMC2939541; doi:10.1186/1471-2318-10-57)

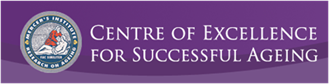

Supplement: Additional file 1 — SHARE Frailty Instrument calculator (females). SHARE-FI calculator - females.zip. [file 1471-2318-10-57-S1.ZIP › SHARE-FI calculator - females/image002.png]

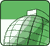

Supplement: Additional file 1 — SHARE Frailty Instrument calculator (females). SHARE-FI calculator - females.zip. [file 1471-2318-10-57-S1.ZIP › SHARE-FI calculator - females/powered-by-spreadsheetconverter.png]
